# GENETIC AND PHARMACOLOGIC PROTEASOME AUGMENTATION AMELIORATES ALZHEIMER’S-LIKE PATHOLOGY IN MICE AND FLIES

**DOI:** 10.1093/geroni/igac059.1664

**Published:** 2022-12-20

**Authors:** Andrew Pickering

**Affiliations:** University of Alabama at Birmingham, Birmingham, Alabama, United States

## Abstract

The proteasome has key roles in neuronal proteostasis, including removal of misfolded and oxidized proteins, presynaptic protein turnover, as well as synaptic efficacy and plasticity. Proteasome dysfunction is a prominent feature of Alzheimer’s disease (AD) (1–3). Artificial impairment of proteasome function can mimic many neurodegenerative phenotypes (4, 5). We report impaired proteasome function to represent an early-stage marker of AD preceding many other markers of the disease. Significantly, we show that prevention of proteasome dysfunction by genetic manipulation in fly and cell culture models of AD delays mortality, cell death, and cognitive deficits. We developed a transgenic mouse with neuronal-specific proteasome overexpression which, when crossed with a mouse model of AD showed reduced mortality and cognitive deficits. To establish translational relevance, we developed a set of novel TAT-based proteasome-activating peptidomimetics. These agonists stably penetrate the blood-brain-barrier and enhance 20S as well as 26S proteasome activity. We show that treatment with these agonists protects against cell death in a cell culture model of AD as well as both cognitive decline and mortality in fly and mouse models of AD. The protective effects observed from proteasome overexpression in our models appear to be driven at least in part by increased turnover of the amyloid precursor protein (APP) by the proteasome. We conclude that the proteasome plays an important role in AD progression. Furthermore, augmentation of proteasome function is protective against AD-like pathogenesis in diverse models of the disease, representing a new therapeutic target for treatment of AD.

